# Unlocking the molecular mechanisms of bamboo-based remediation of hazardous contaminants: macromolecular interactions, transport pathways, and predictive approaches

**DOI:** 10.3389/fpls.2026.1940350

**Published:** 2026-10-05

**Authors:** Muhammad Hayder Ali, Muhammad Ashir Hameed, Ma Cong, Aqsa Malik, Qibin Chen, Shiliang Liu

**Affiliations:** 1 College of Landscape Architecture, Sichuan Agricultural University, Chengdu, China; 2 Institute of Soil and Environmental Sciences, University of Agriculture, Faisalabad, Pakistan; 3 College of Resource Sciences and Technology, Sichuan Agricultural University, Chengdu, China; 4 Department of Botany, University of Narowal, Narowal, Pakistan

**Keywords:** bamboo, heavy metals, lignocellulose, membrane transporters, organic pollutants, phytoremediation, structure-function relationship

## Abstract

Bamboo presents an unusual combination of rapid biomass production and diverse molecular mechanisms for capturing, transforming, and sequestering hazardous contaminants. However, the existing investigations have predominantly focused on bulk removal efficiencies and tissue-level accumulation profiles, whereas the molecular determinants governing these processes remain critically underexplored. This review systematically synthesizes the structure-function relationships of key bamboo-derived macromolecules including cellulose, hemicellulose, lignin, membrane transport proteins such as heavy metal ATPases (HMAs), ATP-binding cassette (ABC) transporters, natural resistance-associated macrophage proteins (NRAMPs), and zinc-regulated/iron-regulated transporter-like proteins (ZIPs), as well as oxidative enzymes such as laccases and peroxidases. Specific emphasis is placed on the molecular features that drive contaminant adsorption, transport, sequestration, and degradation. Particular attention is given to surface functional groups, aromatic domains, conserved transporter motifs, and catalytic metal centers. A critical appraisal of the current literature reveals substantial knowledge gaps, particularly in molecular-weight distributions, three-dimensional conformational states, binding affinities, and direct physicochemical interactions between bamboo-derived macromolecules and hazardous pollutants. To address these limitations, we propose an integrative framework combining proteomics, AI-assisted structure prediction, molecular docking, molecular dynamics (MD) simulations, and quantitative binding assays to uncover the molecular basis of bamboo-pollutant interactions. Future breakthroughs will require the synergistic integration of rigorous structural characterization and direct interaction studies to move from descriptive associations toward predictive structure-function relationships. By consolidating dispersed knowledge and outlining prioritized research agendas, this review furnishes a conceptual framework to advance bamboo-based remediation from descriptive observational studies toward predictive, mechanism-driven strategies for hazardous contaminant management and environmental risk reduction.

## Graphical Abstract

**Figure:**
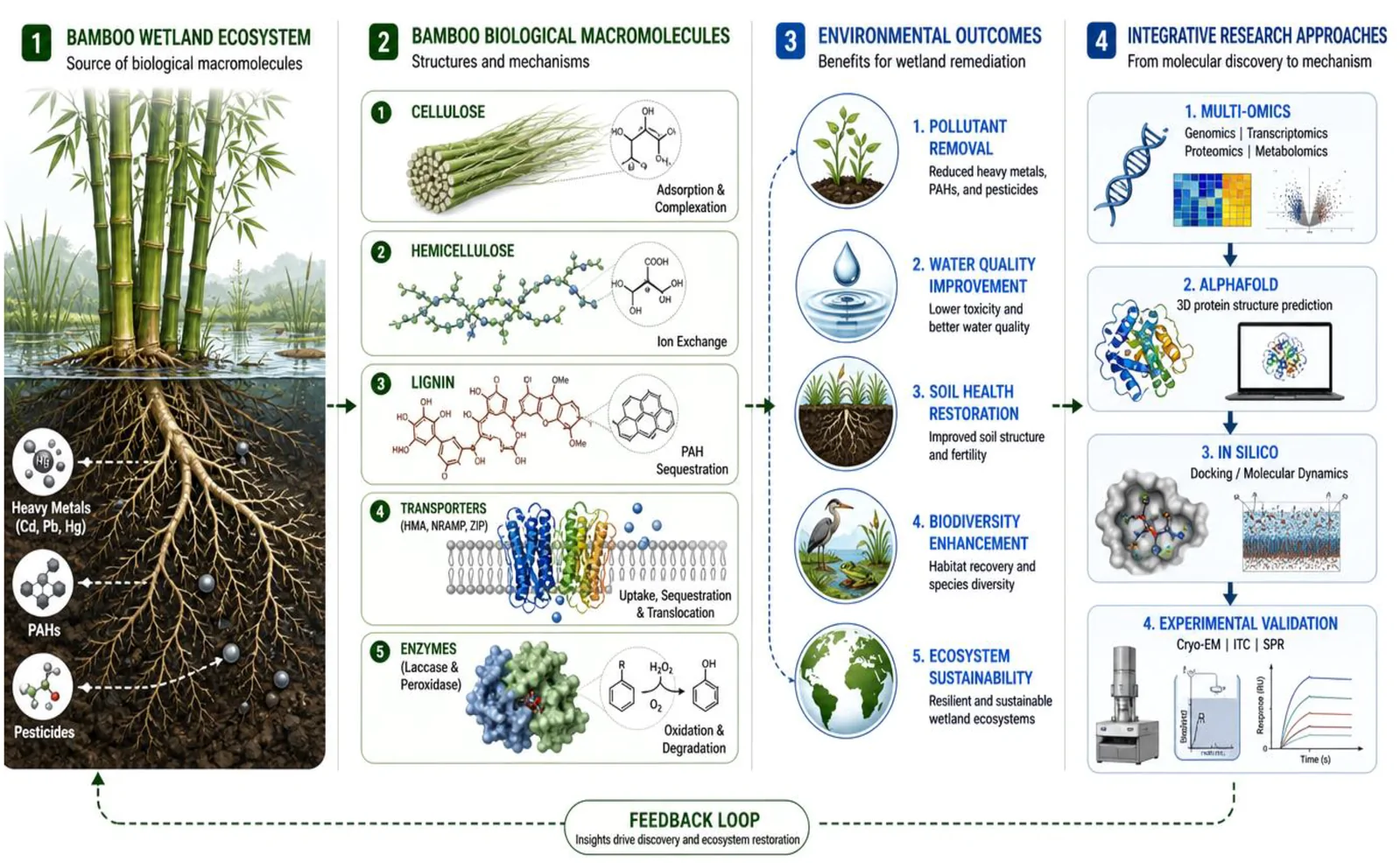
GRAPHICAL ABSTRACT

## Highlights

- Molecular framework for bamboo remediation via cellulose, hemicellulose, lignin, transporters, enzymes.
- Structure–function relationships in contaminant binding/degradation critically evaluated.
- Major gaps: molecular weights, 3D structures, direct kinetics data lacking.
- Integrative tools (proteomics, AI, MD) proposed for mechanistic validation.
- Advocates shift from descriptive to predictive, mechanism-driven strategies.

## 1 Introduction

Contamination of soil and water systems by industrial discharges, agricultural runoff, mining activities, and improper waste disposal poses persistent threats to environmental quality and ecosystem sustainability (Chakraborty et al., 2023). These systems can accumulate diverse contaminants, including toxic heavy metals such cadmium (Cd), lead (Pb), mercury (Hg), chromium (Cr), copper (Cu) and arsenic (As); organic pollutants including polycyclic aromatic hydrocarbons (PAHs), polychlorinated biphenyls (PCBs), pesticides, and pharmaceuticals; and emerging contaminants such as microplastics and polyfluoroalkyl substances (Sall et al., 2020; Xu et al., 2024). The persistence, toxicity, and bioaccumulation potential of these contaminants have driven the development of innovative remediation strategies. Among them, phytoremediation has attracted considerable attention due to its cost-effectiveness, environmental sustainability, and ability to remove, immobilize, or transform contaminants through plant-mediated processes (Akhtar et al., 2021; Priyadarshanee et al., 2022). In addition to reducing contaminant loads, it regulates contaminant mobility, bioavailability, and transformation pathways while minimizing disturbance to affected environments.

A wide range of plant species has been investigated for contaminated soils and waters through mechanisms such as phytoextraction, rhizofiltration, phytostabilization, phytodegradation, and rhizodegradation, thereby facilitating the removal, immobilization, or transformation of diverse contaminants (Ahmad, 2024). Emergent macrophytes such as *Typha latifolia* and floating species such as *Salvinia molesta* are widely used because of their ability to accumulate nutrients and contaminants, whereas woody species such as willow and poplar are valued for their extensive root systems, which promote contaminant stabilization and long-term site restoration (Lima Filho et al., 2025; Tziourrou and Golia, 2025). Among these species, bamboo represents a promising system for investigating molecular mechanisms of contaminant immobilization, transformation, and detoxification owing to its rapid growth, high biomass production, and abundance of structurally diverse biological macromolecules, including cellulose, hemicellulose, lignin, transport proteins, and oxidative enzymes (Mohsin, 2023; Rimal, 2023; Zhang, 2025). These attributes have stimulated extensive research into the use of bamboo for environmental remediation. Existing studies have primarily focused on macroscopic indicators of remediation performance, including contaminant removal efficiency, tissue-specific accumulation, translocation patterns, and physiological responses to pollutant exposure (Uturunco et al., 2024; Das et al., 2025). In contrast, relatively few investigations have examined the biological macromolecules responsible for these processes or explored how their structural characteristics govern contaminant interactions and fate (Rani et al., 2023).

The remediation potential of bamboo is closely linked to several structurally and functionally distinct biological macromolecules. Cellulose is a polymer of β-(1-4)-linked D-glucose units that constitute 40-50% of the dry bamboo biomass, provides abundant hydroxyl groups and a structural framework that can influence interactions with contaminants (Behera et al., 2025). Hemicellulose comprises a heterogeneous group of branched polysaccharides that interact with cellulose microfibrils and contributes to the physicochemical properties and ion-exchange characteristics of the cell wall (Zhu et al., 2022). Lignin, a heterogeneous aromatic polymer, contains phenolic and other oxygen-containing functional groups as well as hydrophobic aromatic domains that may facilitate interactions with organic and inorganic contaminants (Camargos et al., 2025a). At cellular level, membrane transport proteins including heavy metal ATPases (HMAs), ATP-binding cassette (ABC) transporters, natural resistance-associated macrophage proteins (NRAMPs), and zinc-regulated/iron-regulated proteins (ZIPs) facilitate metal ion movement across root membranes through energy-dependent transport systems. In addition, oxidative enzymes such as laccases and peroxidases provide potential pathways for the transformation of organic contaminants (Rashid et al., 2023; Rusch et al., 2023), although direct evidence for their endogenous activity in bamboo remains limited.

Unlike previous bamboo remediation reviews that primarily summarize contaminant removal efficiencies, biomass-derived adsorbents, or phytoremediation performance, this review proposes a molecular-level framework for understanding hazardous contaminant fate and transformation through bamboo-derived biological macromolecules. It critically examines how the molecular architecture of cellulose, hemicellulose, lignin, membrane transport proteins, and oxidative enzymes may influence contaminant adsorption, transport, sequestration, and degradation. Furthermore, the review integrates emerging omics, structural and computational approaches, including proteomics, AI-assisted structure prediction, molecular docking, and molecular dynamics (MD) simulations, to establish a framework for linking macromolecular structure with remediation function. To maintain a clear mechanistic focus, this review emphasizes bamboo-derived biological macromolecules and does not focus on small molecular components such as humic substances, phenolic mediators, and organic acids unless directly involved in macromolecular interactions. This review considers both soil and water associated remediation systems, with emphasis on the molecular processes underlying contaminant fate in bamboo-based remediation. Evidence from non-bamboo or model systems is clearly distinguished from evidence obtained directly from bamboo, and its relevance and limitations are discussed accordingly.

## 2 Structural characteristics of bamboo-derived macromolecules governing contaminant interactions

Bamboo’s remediation potential is closely linked to its lignocellulosic architecture, which is primarily composed of cellulose, hemicellulose, and lignin. Cellulose microfibrils contain abundant hydroxyl groups that contribute to contaminant binding and provide sites for chemical modification (Ivbanikaro et al., 2023; Huang et al., 2026b). Hemicellulose comprises a heterogeneous network of branched polysaccharides, which exhibit greater chemical accessibility and participate in ion-exchange and adsorption processes. Lignin, a complex aromatic polymer contains phenolic and oxygen-containing functional groups that facilitate interactions with organic and inorganic contaminants (Lou et al., 2023). These macromolecules are interconnected through lignin-carbohydrate complexes (LCCs) and contribute to the structural organization of bamboo cell walls. Furthermore, the multilayered and anisotropic architecture of bamboo influences the accessibility of reactive sites and the diffusion of contaminants within the lignocellulosic matrix, thereby affecting contaminant binding, transport, and sequestration processes (Huang et al., 2026b).

### 2.1 Cellulose

Cellulose is the most abundant structural polysaccharide in bamboo cell wall. It consists of linear chains of β-(1→4)-linked D-glucose units, each containing hydroxyl groups at the C2, C3, and C6 positions. These hydroxyl groups form an extensive network of intramolecular and intermolecular hydrogen bonds that contribute to structural stability and organization of cellulose microfibrils. Intramolecular hydrogen bonds, primarily O3–H···O5′ and O2–H···O6′ interactions, stabilize individual glucan chains, whereas intermolecular hydrogen bonds, such as O6–H···O3′, promote the association of adjacent chains into highly ordered crystalline domains (Rashid et al., 2023). Cellulose chains are further organized into sheets through intermolecular associations, forming microfibrils with alternating crystalline and amorphous regions. The crystalline regions are highly ordered and extensively hydrogen-bonded, whereas the amorphous regions are less ordered and generally more accessible to water, chemical reagents, and contaminants. The degree of polymerization of bamboo cellulose typically ranges from 5,000 to 15,000 glucose units, corresponding to molecular weights frequently exceeding 800 kDa (Prasad et al., 2021). In contaminant-removal processes, cellulose hydroxyl groups can provide important interaction sites for metal ions through hydrogen bonding, ion exchange, and, under appropriate conditions. The greater accessibility of functional groups in amorphous regions may favor contaminant interactions compared with highly ordered crystalline regions (Ahmad et al., 2022; Rusch et al., 2023). A comprehensive review by Kaur et al. (2022) demonstrated that the adsorption performance of cellulose-based materials toward Cd^2+^, Pb^2+^, and Cu^2+^ is strongly influenced by the density and accessibility of surface functional groups. This capacity can be further enhanced by introducing carboxyl, amine, thiol, or phosphoryl groups, which increase the availability of metal-binding sites (Kumar and Kumar, 2023).

### 2.2 Hemicellulose

Hemicellulose accounts for approximately 20-30% of the dry weight of bamboo and comprised of arabinoxylans, together with glucomannans and xyloglucans (Bai et al., 2022). Bai et al. (2022) investigated the influence of bamboo cell types on hemicellulose structure in *Phyllostachys pubescens* and demonstrated substantial variations in hemicellulose composition and molecular architecture among parenchyma, fiber, and vascular tissues. Differences in xylan substitution patterns, branching characteristics, and molecular organization making it a highly heterogeneous macromolecule whose physicochemical properties depend strongly on tissue origin. These structural differences may therefore influence the contaminant-binding behavior of hemicellulose fractions derived from different bamboo tissues. While cellulose fibers are long-chain polymers with a degree of polymerization in the thousands, hemicellulose chains are shorter (typically about 100-200) and branched (Zhou et al., 2023). Due to their short length and branching, hemicellulose molecules are more readily soluble in alkali solutions and exhibit greater flexibility than cellulose. Functional groups relevant to contaminant interactions include hydroxyl and carboxyl groups associated with uronic acids, whereas acetyl substituents can modify polymer polarity and accessibility (Kumar and Chang, 2024).

Bamboo hemicellulose fractions enriched in glucuronoarabinoxylan (GAX) contain a β-(1→4)-linked xylopyranose backbone substituted with L-arabinofuranose and 4-O-methyl-D-glucuronic acid side chains which provides multiple potential sites for pollutant interactions (Xue et al., 2023). FTIR analyses have identified abundant oxygen-containing functional groups in bamboo hemicellulose, including hydroxyl (~3425 cm^-^¹) and carboxyl/ester (~1739 cm^-^¹) groups, which serve as potential binding sites for metal cations and can influence the hydrophilicity and adsorption behavior of the polymer (Bagbi et al., 2024). Peng et al. (2018) demonstrated that bamboo hemicellulose functions as a regenerable biosorbent, achieving silver (Ag^+^) adsorption capacities of approximately 42 mg g^-^¹ through ion-exchange, complexation, and electrostatic-attraction mechanisms. The adsorption efficiency was strongly influenced by the availability of uronic acid groups, highlighting the importance of hemicellulose functional-group chemistry in metal binding. Similarly, Shan et al. (2021) compared hemicellulose, cellulose, and lignin together, and showed that these components make distinct but complementary contributions to contaminant adsorption. Hemicellulose, owing to its relatively amorphous structure and abundance of polar functional groups, may favor interactions with ionic contaminants, while interactions among lignin, hemicellulose, and cellulose within lignin-carbohydrate complexes can further influence the accessibility and adsorption behavior of lignocellulosic materials. Another study conducted by Zhang et al. (2020) further demonstrated that pyrolysis can substantially alter the adsorption properties of hemicellulose-derived materials, highlighting how processing-induced changes in functional groups and surface properties can influence contaminant interactions.

### 2.3 Lignin

Lignin is the second-most abundant biopolymer in nature and typically accounts for 20-30% of bamboo dry weight depending upon the species and type of tissues. Lignin is an amorphous heteropolymer formed through the oxidative polymerization of three monolignols: sinapyl alcohol, coniferyl alcohol, and p-coumaryl alcohol, which give rise to syringyl (S), guaiacyl (G), and hydroxyphenyl (H) units, respectively. These phenylpropanoid units are interconnected through ether linkages (β-O-4, α-O-4, 4-O-5) and carbon-carbon linkages (β-β, β-5, 5-5) (Dandash et al., 2025). The resulting three-dimensional network is highly cross-linked and hydrophobic, contributing to the structural integrity of the plant cell wall. Phenolic hydroxyl group, aliphatic hydroxyl groups and carboxyl groups are among the most important functional groups in lignin that contributes to contaminant binding and adsorption processes (Verma et al., 2021). Moreover, the aromatic and phenolic groups of lignin are known for their affinity towards nonpolar organic pollutants including aromatic hydrocarbons such as PAHs, PCBs and pesticides. This binding affinity is attributed mostly to the formation of aromatic interactions in aqueous solution by π-π stacking between the aromatic ring of lignin and the pollutant (Tian et al., 2023). Seidi et al. (2025) further emphasized that the heterogeneous three-dimensional architecture of lignin, together with its phenolic hydroxyl, aliphatic hydroxyl, methoxy, and aromatic functionalities, influences its interactions with metal ions and organic pollutants. Under alkaline conditions, deprotonation of phenolic hydroxyl groups can generate phenolate sites with increased capacity for metal-ion interactions and complexation. In addition to surface interactions, the heterogeneous and amorphous lignin matrix may facilitate contaminant retention through diffusion and entrapment within its polymer network (Guo et al., 2008; Mennani et al., 2024). Furthermore, the porous structure of lignin can influence contaminant retention through size and shape dependent exclusion effects.

Bamboo lignin is a heterogeneous H-G-S (p-hydroxyphenyl-guaiacyl-syringyl) in which β-O-4′ ether linkages constitute the predominant interunit bonds. Its composition and structural characteristics vary among bamboo species, including *Neosinocalamus affinis* and *Bambusa lapidea* (Zhu et al., 2020). Bamboo lignin can also contain γ-acylated structures and hydroxycinnamic acid ester linkages, which may contribute to its structural recalcitrance. For remediation applications, deep eutectic solvent (DES) processing can modify the structural heterogeneity and surface properties of bamboo lignin, potentially improving its accessibility for contaminant adsorption (Li et al., 2025). More broadly, studies of lignocellulose-based biosorbents indicate that aromatic and oxygen-containing functional groups can contribute to contaminant interactions through mechanisms including hydrophobic interactions, π–π interactions, complexation, and electrostatic attraction (Dev Md et al., 2024). These mechanisms provide a comparative basis for understanding how the corresponding structural features of bamboo lignin may contribute to contaminant retention.

### 2.4 Membrane transport proteins

Membrane transport proteins represent a distinct class of biological macromolecules compared with structural polysaccharides. Whereas cellulose, hemicellulose, and lignin contribute primarily to contaminant retention through passive interactions, transport proteins are integral membrane proteins that mediate the uptake, efflux, translocation, and compartmentalization of contaminants across cellular membranes and into intracellular compartments such as vacuoles (Shimada et al., 2021). Transporters implicated in bamboo-contaminant interactions include P-type ATPases, ABC transporters, NRAMPs, and ZIPs as shown in **Figure 1**. Among these, P-type ATPases are ATP-dependent transmembrane pumps that facilitate the transport of metal ions across biological membranes (Rekha et al., 2021). Direct functional studies of contaminant transporters remain limited in bamboo which require comparative evidence from other plants. For example, Zhang et al. (2023) identified and functionally validated Cd-responsive transporters in *Avicennia marina*, while Li et al. (2024b) showed that the rice ABCG transporter OsPDR20 contributes to Cd translocation and homeostasis. These findings provide comparative evidence for investigating analogous transporter-mediated processes in bamboo.

**Figure 1:**
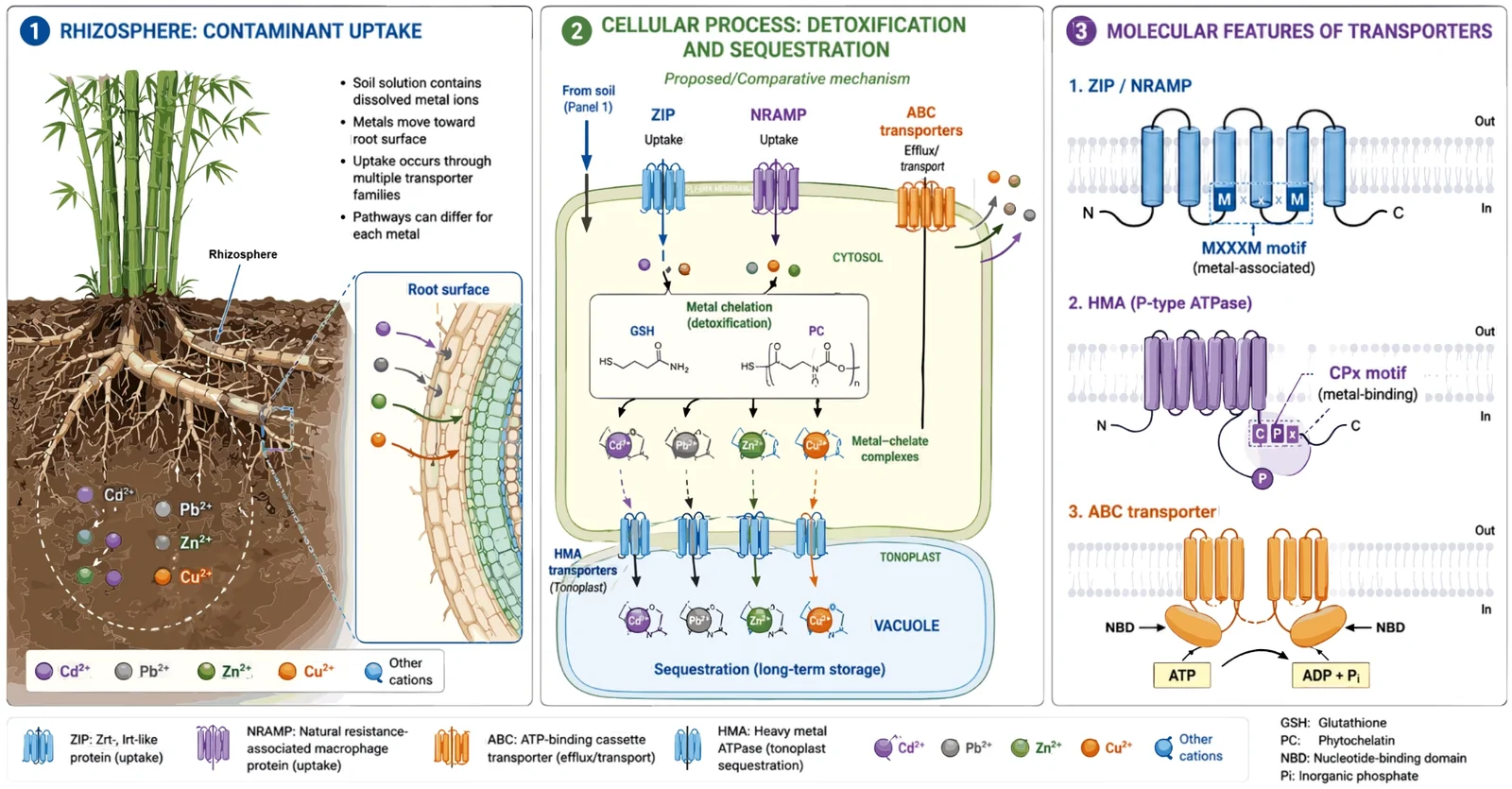
Conceptual framework of proposed and comparative transporter-mediated mechanisms of metal uptake, detoxification, and vacuolar sequestration in bamboo.

P-type ATPases possess conserved phosphorylation domains that drive conformational changes during the transport cycle, enabling alternating access of metal-binding sites across cellular membranes. These transporters also contain characteristic metal-binding motifs, including cysteine-proline-X (CPx) motif, which is critical for metal recognition and transport (Gao et al., 2025). ABC transporters couple ATP binding and hydrolysis to the translocation of diverse substrates, including metal complexes, glutathione-conjugated contaminants, and xenobiotics. Structurally, they contain two transmembrane domains and two nucleotide-binding domains responsible for substrate translocation and ATP hydrolysis, respectively. Similarly, members of NRAMP mediate proton-coupled transport of divalent metals such as Fe²^+^, Mn²^+^, Cd²^+^, and Pb²^+^, whereas ZIP proteins facilitate the transport of Zn²^+^, Fe²^+^, and other divalent metals and contain conserved metal-binding motifs (Khan et al., 2020; Yang et al., 2025a). Their transmembrane architecture and conserved metal-binding motifs are important determinants of substrate recognition and transport. Multidrug and toxic compound extrusion (MATE) transporters represent another transporter family potentially relevant to xenobiotic efflux. They generally function as proton-coupled antiporters and facilitate the extrusion of diverse organic compounds (Liu et al., 2016; Kar et al., 2022). Miyauchi et al. (2017) have shown that structural and functional analysis revealed substrate transport is driven by conformational changes triggered by protonation of a conserved acidic residue. However, the specific contribution of MATE transporters to contaminant transport and remediation in bamboo remains largely unresolved.

### 2.5 Oxidative enzymes

Oxidative enzymes of bamboo, primarily laccases and peroxidases, represent an important class of enzymatic macromolecules with potential roles in contaminant transformation. Unlike structural polysaccharides, these enzymes function as biocatalysts capable of oxidizing a broad range of organic substrates (Xia et al., 2024). Laccases are multicopper glycoproteins, typically ~50–100 kDa, containing four copper atoms organized into T1 and T2/T3 catalytic centers. The T1 copper site accepts electrons from substrates, whereas the trinuclear T2/T3 catalyzes the reduction of O_2_ to H_2_O (Xia et al., 2024). Substrate binding sites in laccases are usually hydrophobic and are specific for aromatic compounds like phenols, anilines, and PAHs. In comparative lignocellulosic and enzymatic systems, oxidation can generate intermediates that subsequently undergo polymerization, coupling, or other transformation reactions; whether analogous processes occur during contaminant transformation by endogenous bamboo enzymes remains to be established. Peroxidases are heme-containing enzymes whose catalytic activity involves hydrogen peroxide-dependent formation of high-valent iron intermediates capable of oxidizing diverse organic substrates (Kaur, 2016). Their catalytic activity is mediated by an iron protoporphyrin IX active site that reacts with hydrogen peroxide to generate a highly reactive iron-oxo intermediate capable of oxidizing a wide range of organic substrates. Li et al. (2020) performed a genome-wide analysis of laccase genes in Moso bamboo identified 23 genes (PeLAC1–PeLAC23). PeLAC10 was upregulated under abiotic stress conditions, suggesting a potential role in stress adaptation. However, its functional contribution to contaminant transformation in bamboo remains to be experimentally established. Li et al. (2016) reported enhanced peroxidase activity in Moso bamboo seedlings under Cd stress, indicating the involvement of POD in the plant response to metal exposure. These observations support the involvement of oxidative enzymes in bamboo stress responses but do not by themselves establish direct degradation or transformation of organic contaminants by endogenous bamboo enzymes.

### 2.6 Analytical characterization of bamboo-derived macromolecules

Studies relying solely on colorimetric or classical analytical methods, without accompanying structural or molecular weight characterization, were interpreted cautiously when assessing structure-function relationships. Reliable identification and characterization of bamboo-derived macromolecules require analytical techniques capable of confirming molecular identity, purity, and molecular weight (Rani et al., 2023). Size-exclusion chromatography including gel permeation chromatography can be used to determine molecular-weight distributions polydispersity (Dos Santos et al., 2015). Fourier-transform infrared (FTIR) spectroscopy provides valuable information on functional groups and peak shifts associated with contaminant binding. Nuclear magnetic resonance (NMR) spectroscopy provides detailed structural characterization, including solid-state ¹³C NMR for polysaccharides and two-dimensional ¹H-¹³C correlation spectroscopy for lignin. X-ray diffraction (XRD) analysis is widely used to determine cellulose crystallinity and evaluate structural changes associated with remediation processes. Additional analytical techniques provide valuable mechanistic insights into contaminant interactions. Scanning electron microscopy coupled with energy-dispersive X-ray spectroscopy (SEM–EDS) can be employed to examine surface morphology and map contaminant distribution, whereas X-ray photoelectron spectroscopy (XPS) enables the characterization of surface chemical states and metal coordination environments. Isothermal titration calorimetry (ITC) provides thermodynamic information on macromolecule-contaminant interactions, including binding stoichiometry, affinity constants, enthalpy, and entropy changes (Bagbi et al., 2024). Surface plasmon resonance (SPR) can complement these approaches by quantifying interaction kinetics. Collectively, these approaches provide the structural, physicochemical, and mechanistic information necessary to establish robust macromolecule-contaminant structure-function relationships.

This review prioritizes studies that provide direct evidence of macromolecular structure, function, or contaminant interactions. Consequently, transcriptomic, metabolomic, phylogenetic, genomic, and routine bioinformatic studies were considered primarily as supporting evidence for structural or functional validation at the macromolecular level. Similarly, machine learning approaches were included when they contributed to macromolecular structure prediction, interaction analysis, or experimentally testable mechanistic hypotheses.

## 3 Molecular mechanisms of contaminant interactions with bamboo-derived macromolecules

The ability of bamboo to interact with and remediate environmental contaminants can be understood through five interrelated molecular processes: adsorption and complexation, hydrophobic interactions, transport-mediated sequestration, enzymatic transformation, and interactions within the lignocellulosic matrix. However, the strength of experimental evidence supporting these processes differs substantially among bamboo systems. Some mechanisms have been directly demonstrated using bamboo tissues or isolated bamboo-derived macromolecules, whereas others are supported mainly by evidence from non-bamboo plants, purified biomaterials, or related biological systems. Therefore, cross-species findings are considered here as mechanistic evidence that may inform hypotheses for bamboo rather than as direct evidence of bamboo-specific function unless experimentally validated in bamboo. General mechanistic parameters and evidence derived primarily from non-bamboo and model systems are summarized in **Table 1**.

**Table 1:** General mechanistic framework for macromolecule–contaminant interactions (Evidence from non-bamboo and model systems).

Mechanism	Macromolecule class	Functional groups/motifs	Contaminant types	Key parameters	Environmental factors	Evidence basis (system studied)	Reference
Adsorption & Complexation	Cellulose	Equatorial –OH (C2, C3, C6); H-bonds	Cd^2+^, Pb^2+^, Cu^2+^, Ni^2+^, Zn^2+^, Co^2+^	Binding capacity 0.1–0.5 mmol/g; Langmuir KL 0.01–0.1 L/mg	pH optimum 5–7; endothermic; affinity Cu^2+^>Pb^2+^>Cd^2+^>Ni^2+^>Zn^2+^ (amorphous cellulose)	Cellulose and chemically modified cellulose (non-bamboo)	(Gurgel et al., 2008)
Adsorption & Complexation	Hemicellulose	–OH, –COOH (uronic acids), –OCH_3_, acetyl	Heavy metals; cationic dyes (methylene blue, crystal violet)	Binding capacity 0.202–0.356 mmol/g; log K 3–6	pH optimum 4–6; Ca^2+^/Mg^2+^ compete; affinity Pb^2+^>Cu^2+^>Cd^2+^>Zn^2+^	Generic low-cost biosorbent (non-bamboo)	(Reddad et al., 2002)
Adsorption & Complexation	Lignin	Phenolic –OH; aliphatic –OH; –OCH_3_; aromatic rings	Heavy metals (soft acids: Hg^2+^, Pb^2+^, Ag+); oxyanions (CrO_4_^2-^, AsO_4_^3-^)	Binding capacity 0.2–0.8 mmol/g	pH >7 favors phenolate; hard metals bind weakly, soft metals bind strongly via cation-π	Generic biomaterials text; Kraft lignin review (non-bamboo)	(Srivastava and Goyal, 2010; Ge and Li, 2018)
Ion Exchange	Hemicellulose, poly-acids	–COO-; –SO_3_- (rare in bamboo); –O–PO_3_^2-^ (if phosphorylated)	Cationic metals; cationic organics (paraquat, diquat)	Cation exchange capacity 0.5–1.5 meq/g; selectivity coefficients 10–100	Selectivity Cs^+^>Rb^+^>K^+^>Na+>Li^+^ (monovalent); Ba^2+^>Sr^2+^>Ca^2+^>Mg^2+^ (divalent)	Generic agricultural biomass (non-bamboo)	(Nurchi and Villaescusa, 2008; Zeaiter, 2024)
Hydrophobic Interactions	Lignin	Aromatic rings (S, G, H units); methoxyl groups; β-O-4 linkages	PAHs, PCBs, organochlorine pesticides, phthalates	Partition coefficient Kd 10^2^–10^5^ L/kg (correlates with log Kow)	Weakens with temperature increase; salinity increases binding	Generic lignin/nano lignin reviews (non-bamboo)	(Camargos et al., 2025a; Lin et al., 2025)
Transport (Uptake)	ZIP family	8 TM domains; MXXXM motif (TM4)	Zn^2+^ (primary), Cd^2+^, Pb^2+^, Mn^2+^, Fe^2+^	Vmax 0.1–10 nmol/mg/min; turnover ~1–10 s-1	External acidic pH favors uptake; Zn^2+^ down-regulates expression	General plant transporter reviews (non-bamboo)	(Tao and Lu, 2022; Yang et al., 2025b)
Transport (Uptake/Efflux)	ABC transporters	2 TM domains; Walker A/B, ABC signature motif	Metal-glutathione/phytochelatin conjugates; organic xenobiotics	Km (ATP) 0.1–1 mM; turnover 1–100 min-1	ATP required; vanadate-inhibited	General Cd-signaling review (non-bamboo)	(Vitelli et al., 2024)
Transport (Sequestration)	HMA (P-type ATPase)	8 TM domains; CPx motif (TM6); DKTGT phosphorylation domain	Cd^2+^, Pb^2+^, Zn^2+^, Co^2+^	Km (Cd^2+^) 0.1–10 µM; turnover 1–100 s^-1^	Vanadate-inhibited; some isoforms CaM-regulated	General HMA review; soybean P1B-ATPases (non-bamboo)	(Chaudhary et al., 2016; Fang et al., 2016)
Transport (Sequestration)	NRAMP family	12 TM helices; conserved Asp/Asn/Ser/His binding site	Fe^2+^, Mn^2+^ (primary); Cd^2+^, Pb^2+^, Co^2+^, Ni^2+^	Km (Fe^2+^) 1–10 µM	More acidic external pH increases uptake	General plant NRAMP reviews (non-bamboo)	(Kanwal et al., 2024; Kozak and Antosiewicz, 2025)
Enzymatic (Laccase)	Laccase (glycoprotein)	3 Cu centers (T1, T2/T3); 4 Cu atoms total	Phenols, anilines, PAHs, lignin monomers, synthetic dyes	kcat 10–1000 s^-1^; Km 1–100 µM	O_2_ required; halides inhibit	Fungal/microbial laccase sources — NOT plant laccase; kinetics may not transfer to bamboo laccase	(Jain and W Ramteke, 2016; Nunes and Kunamneni, 2018)
Enzymatic (Peroxidase)	Peroxidase (heme enzyme)	Heme b; distal His/Arg; proximal His	Phenols, aromatic amines, PAHs, dyes, lignin fragments	kcat 100–1000 s-1; Km (H_2_O_2_) 1–100 µM	H_2_O_2_ required; Ca^2+^ stabilizes	Fungal white-rot lignin/Mn peroxidase (non-bamboo, different peroxidase superfamily than bamboo class III peroxidases)	(Lambertz et al., 2016; Falade et al., 2017)
Molecular Associations	Lignocellulosic matrix	Benzyl ester/ether, phenyl glycoside linkages; ferulate bridges	All contaminant types (combined effects)	Surface area 1–50 m^2^/g (native), 100–500 m^2^/g (delignified)	Alkaline pretreatment removes hemicellulose/lignin, alters binding	Generic lignocellulosic biomass (non-bamboo)	(Escudero-Oñate et al., 2017)

### 3.1 Adsorption and complexation

The interaction of metal ions such as Cd²^+^, Pb²^+^, and Cu²^+^ with bamboo-derived macromolecules involves interconnected processes of adsorption, complexation, and ion exchange. Comparative studies of lignin-based systems indicate that heavy metal removal can involve complexation, ion exchange, electrostatic attraction, and chelation involving oxygen-containing functional groups (Ge and Li, 2018). Cellulose hydroxyl (–OH) and hemicellulose carboxyl (–COOH) groups serve as electron-donating sites that facilitate metal-ion complexation (**Figure 2**). Metal binding increases with pH as deprotonation of these functional groups enhances their affinity for positively charged ions (Kaur et al., 2022). To better understand structural function relationships in bamboo cellulose, Shan et al. (2022) examined the adsorption of tannic acid onto purified bamboo cellulose fibers under different environmental conditions. Their results showed that adsorption was mediated by hydrogen-bond interactions and followed a reversible multilayer adsorption mechanism, with maximum uptake reaching 230 mg g^-^¹ at pH 5. This study provides rare molecular-level evidence linking the functional groups of a bamboo macromolecule directly to its interaction behavior. Hemicellulose can bind more metal ions due to its extra –COOH groups derived from uronic acids compared to cellulose. In addition, lignin binds metal ions using its –OH groups through a deprotonation process in alkaline conditions. Ion exchange process takes place, where contaminant metals replace the naturally occurring counterions of –COOH groups such as H^+^, Ca^2+^, and K^+^ (Xiang et al., 2022). Overall, these studies demonstrate that adsorption and complexation processes are largely attributed by the density, accessibility, and chemical reactivity of hydroxyl, carboxyl, and phenolic functional groups present in cellulose, hemicellulose, and lignin. Variations in pH, functional-group ionization, and macromolecular structure strongly influence contaminant-binding capacity and selectivity (Huang et al., 2026a).

**Figure 2:**
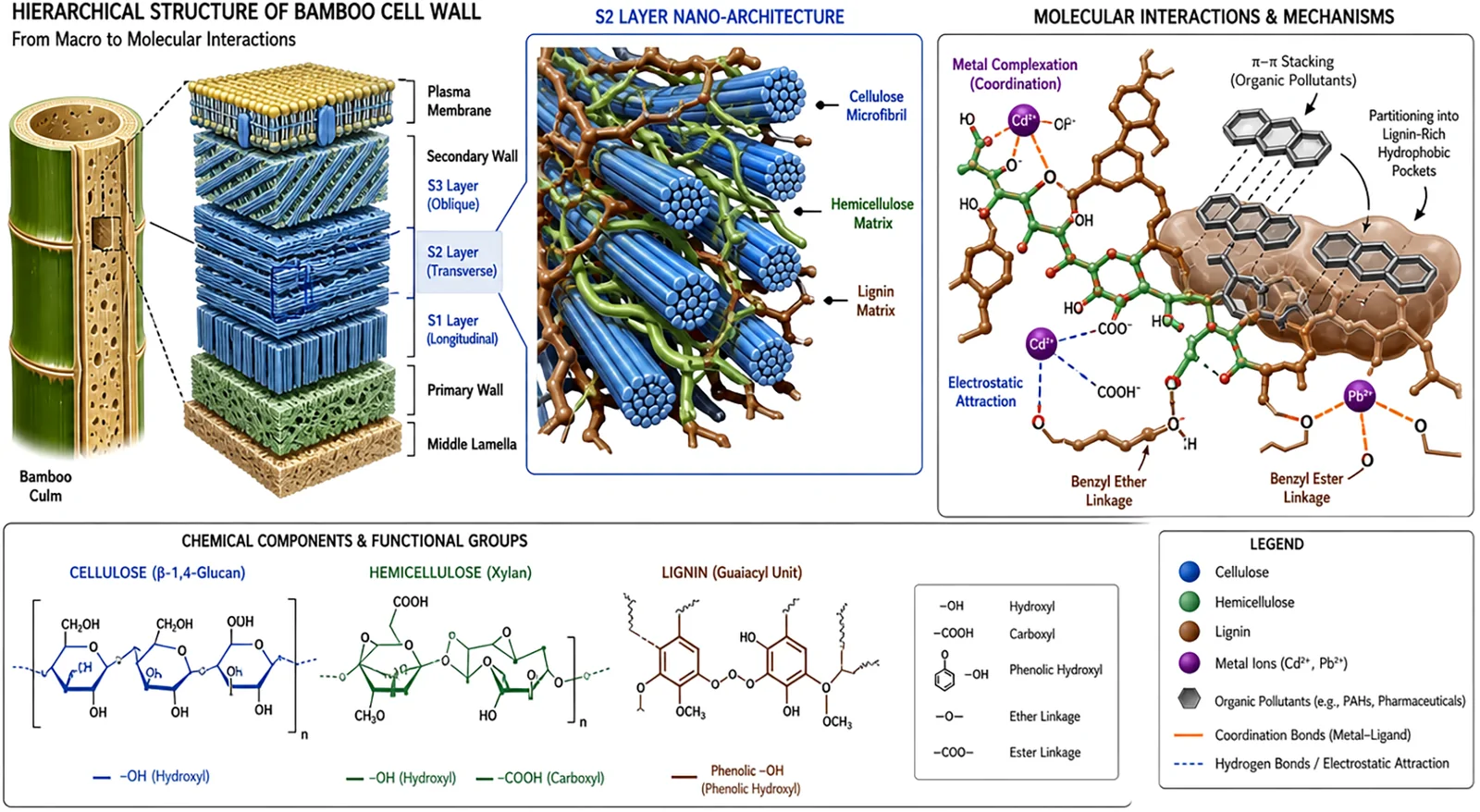
Hierarchical structure of the bamboo cell wall and major molecular mechanisms involved in contaminant interactions. Cellulose, hemicellulose, and lignin provide functional groups and binding domains that facilitate metal complexation, electrostatic interactions, and adsorption of organic pollutants.

### 3.2 Hydrophobic interactions

The retention of nonpolar organic contaminants, including PAHs, PCBs, and some pesticides, can involve hydrophobic interactions and π–π interactions with aromatic domains of bamboo-derived macromolecules. Lignin can contribute substantially to these interactions because its aromatic domains and methoxy-substituted phenyl groups provide hydrophobic and π-electron-rich regions capable of interacting with aromatic organic contaminants. In a comparative study, Seidi et al. (2025) reviewed the chemistry of lignin and condensed tannins and concluded that the abundance of phenolic and aromatic functionalities enables diverse intermolecular interactions, including hydrogen bonding, hydrophobic interactions, π–π stacking, and metal coordination. These structural features provide the molecular basis for the adsorption and immobilization of diverse environmental contaminants by aromatic biopolymers. Furthermore, the amorphous structure of lignin facilitates the diffusion of organic molecules into the polymer matrix, thereby enhancing contaminant retention. In contrast, cellulose and hemicellulose are hydrophilic macromolecules that do not significantly participate in hydrophobic interactions.

Within bamboo-based remediation systems, hydrophobic interactions contribute primarily to the sequestration of organic contaminants rather than heavy metals. Whereas metal removal is largely associated with ion exchange and complexation involving hydroxyl and carboxyl groups, the retention of hydrophobic organic pollutants may be enhanced by aromatic domains within lignin and carbonized bamboo materials through hydrophobic partitioning and π–π electron donor-acceptor interactions (Ahmed et al., 2018; Premlata, 2025). The relative contribution of these mechanisms is influenced by the abundance and accessibility of hydrophobic domains within the lignocellulosic matrix (Sharma et al., 2024; Kumar et al., 2025).

### 3.3 Transport-mediated sequestration

Transport-mediated sequestration is a proposed mechanism contributing to contaminant accumulation and detoxification in bamboo. Membrane transport proteins, including HMAs, ABC transporters, NRAMPs and ZIPs are established components of plant metal transport systems; however, their specific roles in contaminant sequestration in bamboo remain incompletely characterized. ABC transporters operate through an alternating-access mechanism in which substrate binding and ATP hydrolysis induce conformational changes that enable substrate translocation across the membrane (Dutta and Wu, 2014; Rekha et al., 2021). Following cellular uptake, contaminants may be transported into vacuoles by tonoplast-localized HMAs and ABC transporters, thereby reducing their interaction with cytosolic metabolic processes. Vacuolar sequestration represents an important detoxification strategy that can limit contaminant toxicity while facilitating intracellular retention. Yang et al. (2023) investigated Cd toxicity in Moso bamboo (*Phyllostachys edulis*) and found that the plant is highly efficient in Cd uptake, xylem loading, and translocation, with cadmium mainly localized in the root epidermis and pericycle cells. Direct molecular evidence for these specific transporter functions in bamboo remains limited; therefore, mechanistic evidence from other plant species provides comparative context rather than direct confirmation of equivalent functions in bamboo. For example, in rice, OsHMA3 mediates Cd sequestration into root vacuoles, thereby limiting root-to-shoot Cd translocation and grain accumulation (Sasaki et al., 2014). Together, these findings support transporter-mediated vacuolar sequestration as a plausible mechanism of metal homeostasis and provide a comparative basis for investigating the specific roles of HMAs and other transporters in bamboo.

### 3.4 Enzymatic degradation

Bamboo laccases and peroxidases are potential enzymatic contributors to the transformation of organic pollutants. Laccases oxidize phenolic substrates through copper-containing catalytic centers, whereas peroxidases use hydrogen peroxide to oxidize a broad range of aromatic compounds (Datta et al., 2021). Although these catalytic properties provide a plausible biochemical basis for contaminant transformation, direct evidence demonstrating the degradation of environmental contaminants by purified bamboo enzymes remains limited. Unlike passive adsorption, enzymatic degradation permanently transforms contaminants through oxidative reactions (**Figure 3**). These transformations can result in contaminant mineralization or immobilization via oxidative coupling (Lamaming et al., 2022). A recent study by Zhang et al. (2025) investigated bamboo-biochar-supported immobilization of laccase and reported improved thermal tolerance, acid resistance, and reusability compared with free laccase, with 70.9% of the initial activity retained after 10 cycles. Without external mediators, the immobilized laccase system achieved degradation efficiencies of 74.7% for bisphenol A, 85.9% for malachite green, and 94.5% for methyl orange in aqueous solution. These findings demonstrate the potential of bamboo-derived materials to improve the operational stability and reusability of oxidative enzymes for contaminant transformation, but they do not by themselves establish that endogenous bamboo laccases directly degrade these contaminants.

**Figure 3:**
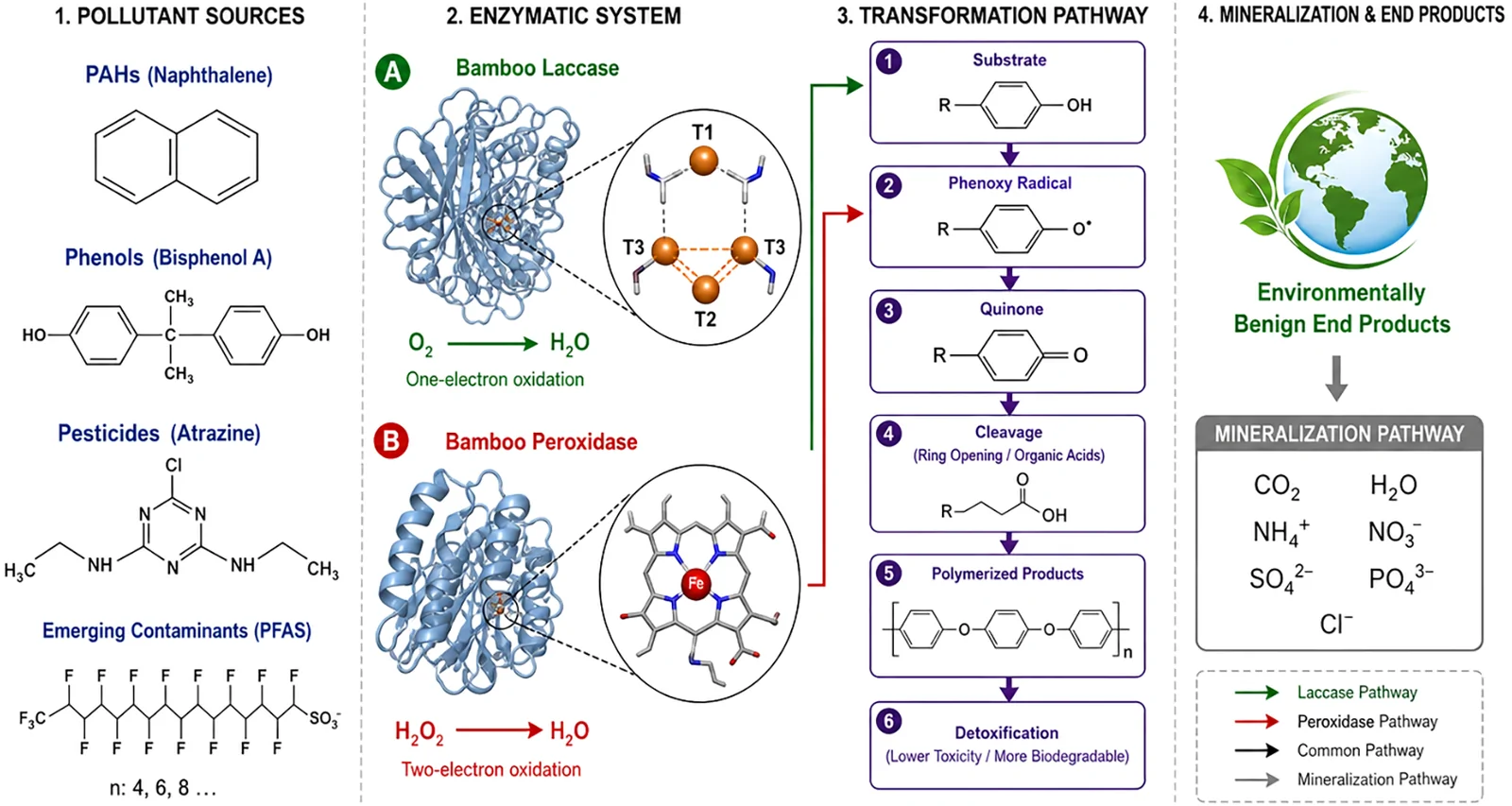
Proposed and comparative mechanisms of organic contaminant transformation by bamboo oxidative enzymes. Laccases and peroxidases may contribute to oxidative transformation through radical-mediated reactions, potentially involving quinone formation, ring cleavage, polymerization, and other transformation processes. These pathways require experimental validation in bamboo systems.

### 3.5 Molecular associations and lignocellulosic cross-linking

In native bamboo tissues, cellulose, hemicellulose, and lignin do not function as separate entities. Rather, they are integrated within a lignocellulosic matrix whose molecular organization attributes the physicochemical properties of the cell wall. Hemicellulose chains are associated with cellulose microfibrils through extensive hydrogen bonding, whereas lignin is linked to hemicellulose through covalent benzyl ester and benzyl ether linkages. The lignocellulosic architecture provides multiple binding domains that can simultaneously interact with different contaminants. For instance, metal ions may be complexed by hemicellulose carboxyl groups, whereas organic pollutants are preferentially retained within the neighboring lignin matrix. However, intermolecular cross-linking reduces the accessibility of certain reactive functional groups, thereby influencing contaminant-binding capacity (Rusch et al., 2023; Su et al., 2025). For example, Wang et al. (2024) demonstrated that bamboo LCCs contain chemically diverse and alkali-stable linkages, including phenyl glycoside and p-coumaric acid-associated structures, which contribute to the structural stability and resistance of the lignocellulosic matrix. Additionally, the study identified the presence of H-lignin-FA-arabinoxylan linkage structures in bamboo LCC. In another study, Lv et al. (2023) characterized lignin and LCCs from bamboo fibers and parenchyma cells and identified substantial differences in lignin composition and LCC linkage patterns between tissue types. These findings indicate that tissue-specific macromolecular architecture may influence the accessibility and chemical reactivity of contaminant-binding sites.

## 4 Integrative approaches to elucidating macromolecular mechanisms

Traditional biochemical approaches provide valuable information on bamboo-derived macromolecules but often cannot resolve the structural and dynamic complexity of their interactions with contaminants. Many membrane-associated proteins cannot be effectively purified for downstream experiments, and some dynamic conformational changes occur at the nanosecond scale. An integrative approach involving both experimental and computational proteomics in conjunction with macromolecule structure and dynamics simulations helps generate meaningful hypotheses about macromolecular functions as presented in **Figure 4**. This section examines the application of these approaches and clarifies the criteria used to define their relevance and inclusion.

**Figure 4:**
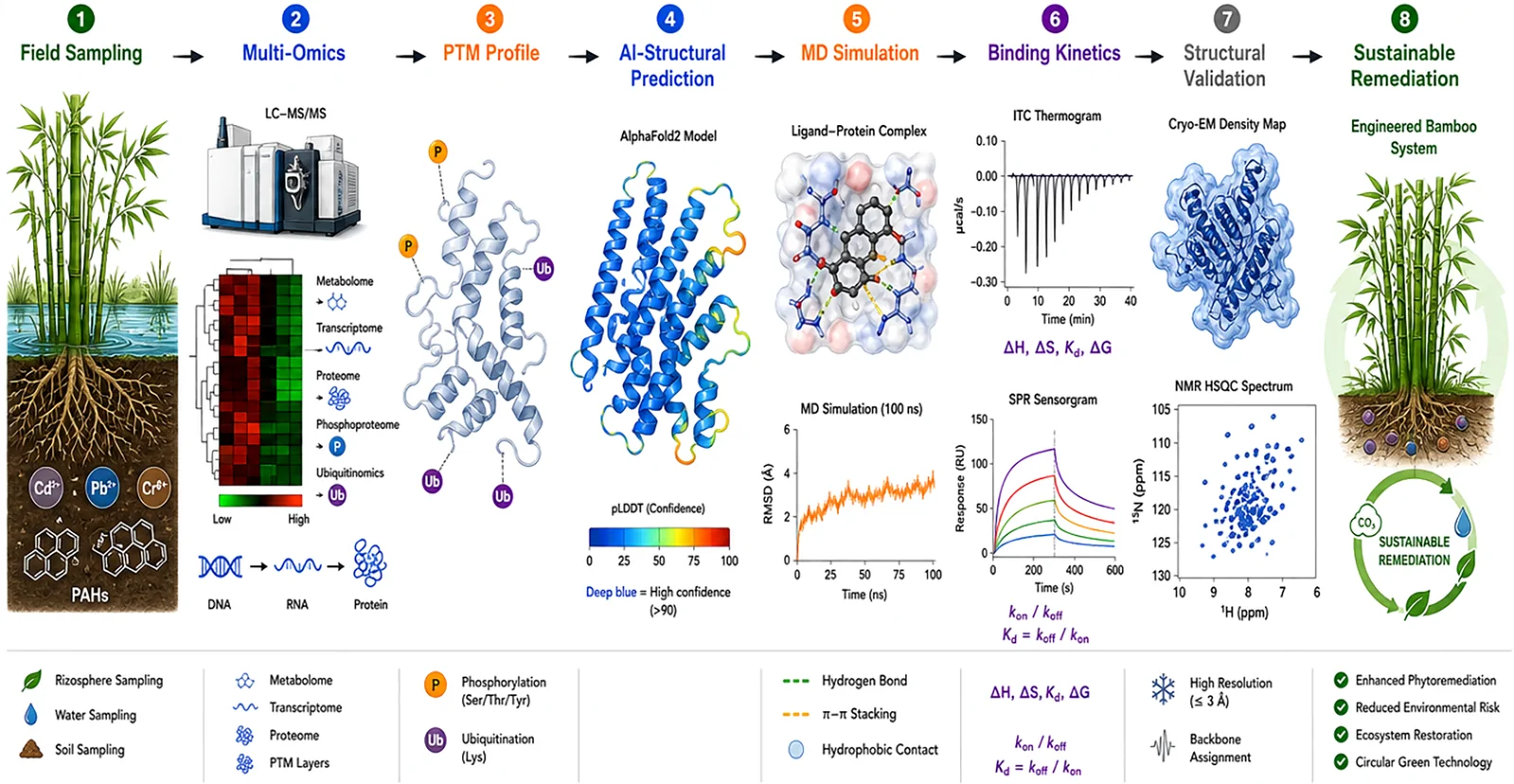
Integrative framework for bamboo macromolecule–contaminant interactions. The workflow combines multi-omics, PTM profiling, AI-assisted structure prediction, molecular dynamics, binding studies, and structural validation to generate and test mechanistic hypotheses linking macromolecular structure with remediation.

### 4.1 Proteomics of contaminant responsive macromolecules

Proteomics enables the identification and characterization of bamboo proteins associated with contaminant response. Candidate membrane transporters, laccases, peroxidases, and other proteins can be identified through proteomic profiling of contaminant-exposed bamboo tissues. Whereas transcriptomics measures change in transcript abundance, proteomics directly identify and quantify proteins within tissues, organelles, and cellular compartments. However, the mere identification of proteins does not provide sufficient evidence to support their mechanistic role in contaminant interactions or remediation processes (Tao et al., 2020; Li et al., 2024a). Proteomics can also identify post-translational modifications (PTMs), such as phosphorylation, glycosylation, ubiquitination, and S-nitrosylation, which influence protein conformation, substrate binding, catalytic activity, stability, intracellular targeting, and stress-response signaling (Zafar et al., 2024). The mechanistic value of proteomics therefore depends on linking protein abundance or PTMs to macromolecular function through complementary activity, binding, structural, or heterologous-expression assays (Delgado et al., 2014; Zafar et al., 2024).

### 4.2 Machine learning-based structure prediction and molecular docking

Machine-learning-based structure prediction, including deep-learning approaches such as AlphaFold2 and RoseTTAFold, can help investigate the three-dimensional architecture of bamboo proteins for which experimentally resolved structures are unavailable (**Figure 4**) (Chen et al., 2024). This is particularly relevant to bamboo transporters and oxidative enzymes, for which structural information remains limited. Computational predictions and docking results should therefore be treated as hypothesis-generating evidence that requires structural, biochemical, or functional validation. For instance, a docking study identifying a putative metal-binding motif in a bamboo HMA transporter becomes mechanistically informative only when supported by subsequent structural, biochemical, or functional validation experiments. Experimental validation may involve approaches such as site-directed mutagenesis and subsequent assessment of mutant protein function (Haneczok and Delijewski, 2021; Khan et al., 2024).

For bamboo remediation, structure-prediction and docking approaches can help generate testable hypotheses for specific molecular questions that cannot currently be resolved experimentally. Candidate HMA, ABC, NRAMP, and ZIP transporter proteins identified or predicted from bamboo genomic or transcriptomic resources could first be structurally modeled to identify conserved domains, transmembrane regions, and putative contaminant-interacting residues. These predictions could then guide targeted mutagenesis, heterologous expression, and transport or binding assays. For bamboo lignin and cellulose, experimentally measured structural descriptors such as lignin S/G ratio, molecular weight, cellulose crystallinity, and degree of polymerization could be incorporated into computational models to investigate how macromolecular architecture influences contaminant interactions. Given the limited bamboo-specific datasets, such predictions should ultimately be evaluated through complementary biochemical, structural, and functional experiments.

### 4.3 Molecular dynamics simulations

In bamboo remediation research, molecular dynamics (MD) simulations can help to address unresolved questions concerning contaminant-macromolecule interactions. However, such applications remain largely prospective because experimentally validated bamboo-specific MD studies are scarce. Therefore, MD simulations should be considered a hypothesis-generating component of an experimental-computational research strategy rather than established evidence of bamboo remediation mechanisms. For candidate bamboo transporters, MD simulations could provide additional insight into dynamic processes associated with contaminant recognition, binding, and translocation, including conformational transitions in ABC transporters and changes in metal-binding regions of ZIP-family transporters (Pietrucci et al., 2021; Mathias et al., 2025). For cellulose, simulations could investigate whether metal adsorption alters hydrogen-bonding networks, structural stability, crystallinity, or the accessibility of additional binding sites (Kou, 2025). Similarly, because lignin is structurally heterogeneous, MD could help characterize contaminant orientation, residence time, and diffusion within lignin-rich domains (Chen et al., 2026). These simulations can therefore complement experimental measurements by generating testable hypotheses about dynamic contaminant-macromolecule interactions. However, because MD simulations depend on model assumptions and parameterization, their predictions require experimental validation before being interpreted as evidence of bamboo-specific remediation mechanisms.

## 5 Current state of evidence for bamboo macromolecule-contaminant interactions

Importantly, the evidence summarized below is not equivalent in strength: studies using intact bamboo, isolated bamboo-derived macromolecules, and non-bamboo model systems provide different levels of mechanistic support. The mechanisms described in Section 3 provide a conceptual framework for understanding contaminant interactions with bamboo-derived macromolecules. However, the extent to which these mechanisms have been experimentally validated varies considerably across the available literature. Therefore, it is important to critically examine the current evidence base, including the types of research approaches employed, the level of macromolecular characterization achieved, and the major methodological limitations that influence mechanistic interpretation.

### 5.1 Overview of existing research approaches

Biomass-based adsorption studies typically employ dried, ground, and sieved bamboo stem or leaf materials in batch adsorption experiments. Although many studies report high removal efficiencies for heavy metals, dyes, and other contaminants, mechanistic evidence is often limited to FTIR-based identification of hydroxyl, carboxyl, and phenolic functional groups. A major limitation of these studies is that bamboo biomass comprises a heterogeneous mixture of cellulose, hemicellulose, lignin, proteins, and other constituents. Without fractionation and purification, the contribution of individual macromolecules cannot be distinguished, making it difficult to attribute contaminant binding to a specific biological macromolecule or establish definitive structure-function relationships. Furthermore, molecular weight characterization of the active components is rarely reported, limiting confirmation of their macromolecular identity and restricting mechanistic interpretation of remediation performance (Zhu et al., 2024a; Das and Sarma, 2025). Unlike bulk biomass studies, Bai et al. (2022) examined hemicelluloses isolated from specific bamboo cell types and demonstrated pronounced structural differences among tissues. Their work illustrates how macromolecule-specific characterization can reveal structural variability that would otherwise remain obscured in bulk biomass analysis.

Living bamboo studies offer an opportunity to investigate contaminant uptake, sequestration, and enzymatic degradation under environmentally relevant conditions. Such studies commonly monitor contaminant accumulation in plant tissues and may include analysis of transporter abundance, enzyme activity, or stress-response biomarkers (Hibakhadeeja et al., 2025). However, because the underlying proteins are rarely purified, structurally characterized, or subjected to direct binding assays, the resulting evidence remains largely correlative. Thus, these studies provide important physiological and ecological evidence but limited direct evidence for specific macromolecule-contaminant interactions (Sharma et al., 2021). Studies employing purified bamboo-derived macromolecules remain relatively uncommon but provide some of the most informative mechanistic evidence available. A limited number of investigations have isolated cellulose, hemicellulose, or lignin and evaluated their contaminant-binding properties using techniques such as FTIR, XRD, NMR, and, in some cases, SEC/GPC for molecular weight determination (Zeng et al., 2024; Negi et al., 2025). These studies provide some of the most direct evidence for macromolecule-specific interactions; however, important gaps remain. Few studies directly compare the binding capacities of different macromolecules from the same bamboo source, even fewer examine synergistic interactions among macromolecules, and mechanistic investigations of transport proteins and enzymes remain largely absent. These knowledge gaps continue to limit a comprehensive molecular-level understanding of bamboo-mediated remediation. Representative experimentally demonstrated evidence from bamboo tissues, isolated bamboo-derived macromolecules, and bamboo-derived functional materials is summarized in **Table 2**.

**Table 2:** Selected bamboo-specific and bamboo-derived evidence for macromolecule-contaminant interactions.

Evidence level	Bamboo species/material	Contaminant/macromolecule/findings	Characterization methods	Reference
Bamboo-derived material	Phyllostachys pubescens biochar	Cd^2+^: Qmax = 14.7 mg/g, with π-electron coordination contributing 54.1–82.6% of Cd^2+^ retention. Pb^2+^: Qmax = 67.4 mg/g, with mineral precipitation contributing 17.2–71.7%; the contribution increased with pyrolysis O_2_ content.	FTIR, SEM-EDS, BET; Langmuir model	(Zhang et al., 2025)
Bamboo-specific direct	Bamboo cellulose fibers	Tannic acid, an organic polyphenol; reversible multilayer adsorption was reported, with hydrogen bonding proposed as an important interaction mechanism. Maximum uptake was 230 mg/g at pH 5.	FTIR-based mechanistic study	(Shan et al., 2022)
Bamboo-specific direct	Bamboo hemicellulose (arabinoxylan)	Ag^+^ adsorption (~42 mg/g) through ion exchange, complexation, and electrostatic interactions; adsorption was associated with the availability of uronic acid groups, and the material showed regeneration potential.	Not reported	(Peng et al., 2018)
Bamboo-specific indirect	*Phyllostachys edulis*, hydroponic	Cd^2+^ uptake, xylem loading, and translocation were observed, with Cd mainly localized in root epidermal and pericycle cells. Transcriptomic responses support involvement of metal-transport processes, but direct interactions between specific transporter proteins and Cd^2+^ were not demonstrated.	Physiological + transcriptomic	(Yang et al., 2023)
Bamboo-derived material	*P. edulis* lignin-derived porous material	Lignin-derived porous material from *P. edulis* was evaluated for removal of aromatic pollutants, including phenanthrene	GPC, FTIR, 2D-NMR HSQC, XRD, fluorescence quenching	(Marangon et al., 2025)
Bamboo-derived material	Bamboo biochar-immobilized laccase	Bisphenol A, malachite green, and methyl orange were degraded with efficiencies of 74.7%, 85.9%, and 94.5%, respectively. No mediator was required, and 70.9% of enzymatic activity was retained after 10 reuse cycles.	Not reported	(Zhang et al., 2017)
Bamboo-derived material	Bamboo culm-derived biochar	Atrazine adsorption was highest for biochar produced at 450 °C; adsorption followed a Langmuir model, while pyrolysis temperature influenced aromaticity and porosity.	Elemental analysis, pHpzc, N_2_ physisorption, SEM, FTIR	(Sbizzaro et al., 2021)

### 5.2 Critical assessment of current evidence and methodological limitations

The principal limitation of the current evidence base is therefore not the absence of remediation studies, but the limited molecular resolution with which their mechanisms have been investigated. Many studies do not establish how molecular weight, polymer architecture, structural heterogeneity, or specific binding sites influence contaminant interactions. This limitation is especially important when comparing cellulose, hemicellulose, lignin, transport proteins, and oxidative enzymes, because differences in molecular structure may strongly influence binding affinity, selectivity, transport, and transformation. Consequently, quantitative molecular characterization and direct interaction measurements are required to move beyond correlations between bulk remediation performance and presumed functional groups.

A major unresolved issue is the lack of molecular-weight characterization of isolated bamboo-derived macromolecules. In many studies, cellulose, hemicellulose, and lignin fractions are evaluated without analysis by size-exclusion chromatography or gel permeation chromatography, making it difficult to determine molecular weight distributions, assess sample purity, or exclude the presence of low molecular weight components (Tolbert et al., 2014; Andreo-Martínez et al., 2021; Rojas et al., 2025). Consequently, uncertainty remains regarding the identity of the macromolecules responsible for contaminant interactions and remediation performance, thereby limiting mechanistic interpretation and comparison among studies. In addition, colorimetric analysis including Folin-Ciocalteu, anthrone, and Bradford reactions yield only overall information about the presence of phenolics, carbohydrates, and proteins (Bastola et al., 2017). These assays provide bulk compositional estimates but cannot establish molecular size, polymer architecture, or structural modifications associated with contaminant binding. For example, the Folin-Ciocalteu assay responds to a wide range of phenolic compounds and cannot distinguish between polymeric lignin and low molecular weight phenolics such as gallic acid or vanillin. Therefore, colorimetric measurements alone provide insufficient evidence for macromolecular characterization and should be complemented by molecular weight determination and structural analysis, such as SEC/GPC and NMR spectroscopy. Similarly, FTIR spectroscopy is frequently used to infer contaminant binding, yet qualitative observations of peak shifts alone rarely provide definitive mechanistic evidence. More rigorous approaches, including quantitative peak analysis, difference spectroscopy, two-dimensional correlation spectroscopy, and appropriate controls, are required to distinguish specific macromolecule-contaminant interactions from non-specific effects or spectral artifacts.

The main deficiency in our understanding lies in the molecular analysis of proteins that participate in the process of contaminants’ removal via transport proteins and enzymes responsible for their breakdown. Although several studies have examined pollutant-induced changes in genes encoding metal transporters and oxidative enzymes, the direct roles of these proteins in contaminant binding, transport, or transformation remain insufficiently validated experimentally (Jin et al., 2023). Currently, only few studies have reported the purification of these proteins and determination of their molecular weight, structure, and binding properties using methods like SEC-MALS, mass spectrometry, protein structure modeling, X-ray crystallography, cryo-electron microscopy, ITC, or SPR (Zhu et al., 2024b; Bhanse et al., 2025; Fan et al., 2026). Similarly, enzyme activity is frequently measured using model substrates, whereas purification and structural characterization of the corresponding enzymes remain relatively uncommon.

The environmental relevance of many experimental studies remains questionable. For example, some investigations evaluate Cd²^+^ adsorption at concentrations as high as 500 mg L^-^¹, whereas environmentally relevant concentrations are often substantially lower (Soda et al., 2021). In addition, the effects of competing ions, dissolved organic matter, and other environmental factors are frequently overlooked. Another limitation is inconsistent reporting and interpretation of binding data. Adsorption studies frequently apply Langmuir, Freundlich, Temkin, or Dubinin–Radushkevich models without adequately justifying model selection. Although qmax is commonly reported, parameters describing binding affinity and favorability, such as KL and RL, are often omitted. Thermodynamic and kinetic parameters are also frequently reported without sufficient consideration of their assumptions and temperature dependence, limiting meaningful comparison among studies (Mabuza et al., 2022; Mahanty et al., 2023).

Despite significant advances in bamboo-based remediation research, the molecular mechanisms of transporter-mediated sequestration and enzymatic degradation remain largely unresolved. Future studies integrating molecular weight determination, structural characterization, and quantitative interaction analysis using techniques such as SEC/GPC, NMR, ITC, SPR, X-ray crystallography, and cryo-EM will be critical for validating these mechanisms and advancing the field from phenomenological observations to molecular-level understanding.

## 6 Challenges and future perspectives for molecularly guided bamboo remediation

The preceding sections outlined the proposed mechanisms by which bamboo-derived macromolecules interact with and remediate environmental contaminants. However, the critical evaluation of the available literature revealed substantial knowledge gaps, particularly when compared with the mechanistic understanding established for other plant species and non-bamboo macromolecular systems. This section identifies the most significant gaps limiting our understanding of bamboo-derived macromolecules and contaminants. It also highlights future research priorities needed to establish definitive structure-function relationships and validate proposed remediation mechanisms. Accordingly, transporter families identified through bamboo transcriptomic or genomic studies should be regarded as candidate components of contaminant transport pathways until their protein-level functions are experimentally validated.

### 6.1 Key knowledge gaps

A major gap in the current literature is the lack of experimentally characterized bamboo transport proteins involved in contaminant remediation. Although transcriptomic studies have identified members of the HMA, ABC, NRAMP, and ZIP transporter families that respond to Cd, Pb, and other metal stresses, their molecular functions remain largely unresolved. Current interpretations of transporter-mediated metal sequestration in bamboo rely primarily on indirect evidence derived from gene expression analysis and physiological responses to contaminant exposure. To date, few studies have pursued heterologous expression, protein purification, molecular weight determination, structural characterization, or quantitative analysis of transporter-contaminant interactions using techniques such as SEC-MALS, X-ray crystallography, cryo-EM, ITC, or SPR. Consequently, direct information regarding transporter structure, substrate specificity, binding affinity, and transport kinetics is lacking, and the extent to which knowledge derived from Arabidopsis, rice, and yeast can be extrapolated to bamboo remains uncertain.

A similarly important knowledge gap concerns the structure-function relationships of bamboo laccases and peroxidases involved in contaminant degradation. Only a few studies exist that describe laccase or peroxidase activity in bamboo extract against artificial models, e.g., using ABTS or guaiacol as substrates, and even fewer describe partial purification of these enzymes. To date, reports describing highly purified bamboo laccases or peroxidases, together with detailed molecular weight determination, structural characterization, and kinetic analysis toward environmental contaminants, remain extremely limited. The few existing measurements of enzyme activity on these pollutants in bamboo extract were made using crude samples where interference by other proteins or cofactors was possible. Like transporters, the biochemical process of degradation mediated by bamboo enzymes has not been experimentally confirmed yet.

MD simulations represent an important but underexplored approach for bamboo-derived macromolecules. They could address unresolved questions concerning how metal binding alters hydrogen-bonding networks in bamboo cellulose, how hydrophobic contaminants interact with lignin domains, and how conformational changes influence contaminant recognition by bamboo transport proteins. Such simulations should be integrated with experimental structural and binding data to generate testable bamboo-specific mechanistic hypotheses.

Most investigations focus on a single macromolecule, typically cellulose, hemicellulose, or lignin, in isolation. As a result, it remains unclear which macromolecule contributes most to the binding of specific contaminants, whether synergistic interactions occur among different macromolecules, or how the supramolecular organization of the native lignocellulosic matrix influences contaminant retention relative to its individual components. Comparative studies using multiple macromolecular fractions isolated from the same bamboo source, together with reconstructed lignocellulosic systems, are therefore needed.

Limited molecular weight characterization of bamboo-derived lignins and polysaccharides is another important knowledge gap. SEC/GPC analyses are reported in only a small proportion of published studies, leaving the molecular weight distributions of extracted macromolecules largely unknown. In addition, polydispersity indices (Đ = Mw/Mn) are rarely determined, preventing assessment of sample heterogeneity and its potential influence on contaminant-binding behavior. For example, highly polydisperse lignin samples may contain substantial low-molecular-weight fractions with adsorption properties that differ from those of the dominant macromolecular fraction, complicating the interpretation and comparison of binding studies.

Another underexplored area is the development of predictive models for bamboo macromolecule-contaminant interactions. Although quantitative structure–activity relationship (QSAR) models and related approaches have been used to predict contaminant interactions with organic carbon and lignin from non-bamboo sources, comparable models have not been developed specifically for bamboo-derived macromolecules. As a result, important structural parameters such as the lignin syringyl/guaiacyl (S/G) ratio, cellulose crystallinity index, and degree of polymerization are rarely incorporated into predictive frameworks. Integrating these characteristics into computational models could facilitate the rapid screening of bamboo species, macromolecular fractions, and treatment strategies for contaminant remediation. Collectively, these knowledge gaps highlight the need for a transition from descriptive remediation studies toward mechanistic investigations grounded in macromolecular characterization. The major unresolved bamboo-specific questions and corresponding experimental and computational approaches are summarized in **Table 3**.

**Table 3:** Unresolved bamboo-specific questions addressable by experimental and computational approaches.

Unresolved bamboo question	Approach	What this could reveal for bamboo	Experimental validation needed	References
Which bamboo HMA, ZIP, NRAMP, or ABC transporters have known sequences but unresolved 3D structures?	AlphaFold2/RoseTTAFold structure prediction from published bamboo transcriptome/genome sequences	Candidate transmembrane topology and putative metal-binding pockets (e.g., CPx motif in HMA, MXXXM motif in ZIP) that can be prioritized for experimental work, rather than a generic structure-prediction demonstration	At least one representative bamboo transporter solved experimentally (X-ray or cryo-EM) to benchmark prediction confidence before broader reliance on the models	(Peng et al., 2022; Willems et al., 2023)
Which predicted residues or motifs could be prioritized for site-directed mutagenesis?	Molecular docking of Cd^2+^, Pb^2+^, atrazine, and PAH ligands onto AlphaFold-predicted bamboo transporter structures	A short, testable list of specific binding-pocket residues, rather than a genome-wide or purely descriptive docking exercise	Site-directed mutagenesis of the flagged residues (e.g., Cys→Ser in a predicted CPx motif), with functional/binding assays comparing wild-type and mutant	(Kamerlin et al., 2020)
Which bamboo lignin S/G ratios or cellulose crystallinity parameters are already measured and could be built into predictive models?	MD simulations parameterized with measured bamboo-specific lignin oligomers and cellulose microfibril structure	Whether documented structural variation among bamboo species/tissues (e.g., differing S/G ratios reported for *Neosinocalamus affinis* vs. *Bambusa lapidea*) translates into meaningful differences in contaminant residence time, diffusion, or binding-site accessibility	MD-predicted binding behavior checked against existing experimental adsorption data on bamboo cellulose/lignin for the same or comparable material	(Zhu et al., 2020; Prasad et al., 2021)
Which contaminant–macromolecule systems already have enough experimental binding data to support predictive (QSAR/ML) modeling?	ML/graph-based binding-affinity prediction trained on the existing pool of bamboo and closely related lignocellulose binding data	Given how few bamboo-specific binding datasets currently exist, this is realistically a near-term, case-by-case tool rather than a generalizable trained model	New experimental binding isotherms (ITC or batch sorption) generated specifically for held-out contaminant–macromolecule pairs, to test whether predictions extrapolate beyond the current small training set	(Peng et al., 2018; Nascimento et al., 2022; Shan et al., 2022)
What contaminant-responsive post-translational modifications remain uncharacterized in bamboo transport proteins and enzymes?	Proteomics/PTM mapping (LC-MS/MS, IMAC phosphopeptide enrichment) of bamboo tissue under contaminant exposure vs. control	Candidate phosphorylation/glycosylation sites on transporters, laccases, and peroxidases that could regulate contaminant response-currently unstudied in bamboo, unlike in several model species	Site-directed mutagenesis of the modified residue (e.g., phosphomimetic Glu/Asp substitution) with activity or binding comparison to wild type	(Hamzah et al., 2016; Qiu et al., 2020; Gupta et al., 2023)

### 6.2 Future research priorities

A major research priority is to establish the molecular basis of bamboo transport proteins involved in contaminant uptake, translocation, sequestration, and detoxification. Attention should be directed toward HMA, ABC, NRAMP, and ZIP transporters, whose involvement in metal stress responses has been suggested by transcriptomic studies but remains to be validated at the protein and functional levels. Heterologous expression and purification followed by structural and functional characterization could establish the molecular properties of these candidate transporters. Approaches such as cryo-EM, X-ray crystallography, ITC, and SPR could subsequently resolve structural features, binding properties, and transport-related interactions.

Bamboo plant laccases and peroxidases should undergo purification studies for structural and kinetic investigations. The proteins can be identified and cloned through mass spectrometry (LC-MS/MS peptide sequencing). It is important to determine kinetic parameters (*kcat*, *Km*, and *kcat*/*Km*) using both model substrates such as ABTS and guaiacol and environmentally relevant contaminants, including chlorophenols, bisphenol A, anilines, and PAHs. The pH optima, temperature optima, and stability profiles of these enzymes should also be determined. X-ray structures and cryo-EM structures will give information about the substrate pocket structure and metallo centers. Site-directed mutagenesis of active site residues will identify the factors governing the specificity for substrates.

Another important research priority is the implementation of direct binding studies between purified bamboo-derived macromolecules and contaminants. Techniques such as isothermal titration calorimetry (ITC) can quantify binding stoichiometry, affinity, and thermodynamic parameters of macromolecule–contaminant interactions. For weaker interactions, fluorescence- or NMR-based titration methods can offer complementary insights into binding behavior. Surface plasmon resonance (SPR) could complement equilibrium binding measurements by quantifying association (*k*on) and dissociation (*k*off) kinetics. Compared with conventional adsorption isotherms, these approaches provide more direct evidence of macromolecule-contaminant interactions and are essential for establishing robust structure–function relationships at the molecular level. MD simulations could provide atomistic insights into the mechanisms and dynamics of contaminant interactions with bamboo-derived macromolecules. Initially, the MD simulation studies may involve the use of representative model systems that mimic the most important structural characteristics of bamboo-derived macromolecules. For studying conformational transitions and the mechanism of interaction, homology models/AlphaFold-predicted structure of the transmembrane protein can be useful.

The development of modified bamboo-derived macromolecules represents another promising research direction for enhancing remediation performance. Consequently, rational chemical and biological modifications may improve adsorption capacity, selectivity, and binding kinetics. For cellulose, promising approaches include TEMPO-mediated oxidation, phosphorylation, carboxymethylation, and amination, which introduce additional functional groups capable of interacting with contaminants. To establish structure–function relationships, all modified materials should be comprehensively characterized using techniques such as SEC/GPC, FTIR, NMR, and XPS to determine molecular weight distributions, structural integrity, and the extent of functionalization.

Another promising research direction is the development of predictive models for bamboo macromolecule–contaminant interactions. Quantitative structure–activity relationship (QSAR) models could be established using binding data obtained from ITC, SPR, or adsorption experiments involving contaminants with diverse physicochemical properties, including hydrophobicity (log P), molecular weight, polar surface area, and pKa. Molecular docking scores may serve as additional descriptors linking contaminant structure to binding behavior. For bamboo lignin, important model parameters could include the syringyl/guaiacyl (S/G) ratio determined by 2D-NMR HSQC, whereas cellulose-based models may incorporate crystallinity index and degree of polymerization. More advanced machine learning approaches, including random forests, gradient boosting algorithms, and neural networks, may further improve predictive accuracy when trained on sufficiently large and diverse datasets of macromolecule–contaminant interactions.

Progress in the field would also benefit from greater standardization of analytical characterization and data reporting. Recommended practices include reporting molecular weight distributions and polydispersity by SEC/GPC, providing comprehensive structural characterization using techniques such as FTIR, NMR, XPS, and SEC-MALS, and reporting quantitative binding parameters obtained from direct interaction studies. Comparative analysis before and after contaminant binding should be routinely performed to establish structure–function relationships. For transporter and enzyme studies, direct evidence from purified proteins should complement transcriptomic and gene expression data.

Collectively, these research directions can help advance bamboo remediation studies from descriptive evaluations of bulk biomass to a mechanistic understanding based on well-characterized biological macromolecules. Such advances would strengthen the molecular basis of bamboo-mediated remediation and facilitate the development of predictive frameworks linking macromolecular structure to contaminant-binding behavior. Ultimately, this knowledge could support the identification of bamboo macromolecular traits associated with enhanced contaminant-binding and remediation capacity and guide the rational development of bamboo-based remediation strategies.

## 7 Conclusion

The wide spectrum of biopolymers present within bamboo confers a range of functional properties relevant to contaminant remediation by means of metal complexation, ion exchange, hydrophobic interactions, membrane transport, and biochemical degradation, among others. Cellulose, hemicellulose, lignin, membrane transport proteins, and oxidative enzymes possess structural features that may contribute to their interactions with contaminants, although the molecular functions of several transporter and enzyme systems remain insufficiently validated in bamboo. Elucidating these structure-function relationships is imperative to predict the effectiveness of any bamboo system and to improve remediation processes. There is currently a large lack of molecular evidence backing many of the proposed processes of remediation involving bamboo biomass. Although much research has been done regarding the use of bamboo for environmental applications, very little is known about the macromolecules themselves, transport proteins, and oxidative enzymes. This includes data concerning molecular size distribution, three-dimensional structure, binding energy, kinetics, and substrate-enzyme interaction parameters. The advancement of these techniques will allow for greater comprehension and comparisons between different studies. Ultimately, these methodologies will enable structure-function relationships to be established, thus facilitating the design of bamboo-based systems for contamination management, optimized bamboo germplasm, and novel biomaterials.
